# How is the evolution of tumour resistance at organ-scale impacted by the importance of the organ for fitness?

**DOI:** 10.1186/s12862-018-1298-7

**Published:** 2018-12-06

**Authors:** Cindy Gidoin, Beata Ujvari, Frédéric Thomas, Benjamin Roche

**Affiliations:** 1 Centre for Ecological and Evolutionary Research on Cancer (CREEC), MIVEGEC, IRD, CNRS, Univ. Montpellier, Montpellier, France; 2 Centre for Integrative Ecology, School of Life and Environmental Sciences, Deakin University, Waurn Ponds, VIC Australia; 3 Sorbonne Université, IRD, UMMISCO, F-93143 Bondy, France; 4 Departamento de Etología, Fauna Silvestre y Animales de Laboratorio, Facultad de Medicina Veterinaria y Zootecnia, Universidad Nacional Autónoma de México (UNAM), Ciudad de México, México

**Keywords:** Trade-off, Metastasis, Cancer risk, Tumorigenesis

## Abstract

**Background:**

A strong variability in cancer incidence is observed between human organs. Recently, it has been suggested that the relative contribution of organs to organism fitness (reproduction or survival) could explain at least a part of the observed variation. The objective of this study is to investigate theoretically the main factors driving the evolution of tumour resistance mechanisms of organs when their relative contribution to organism fitness is considered. We use a population-scale model where individuals can develop a tumour in a key organ (i.e. in which even a small tumour can negatively impact organism fitness), an auxiliary organ (i.e. in which only a large tumour has a relatively significant impact) or both organs because of metastasis.

****Results**:**

Our simulations show that natural selection acts in two different ways to prevent cancer in a key and an auxiliary organs. In the key organ, the strategy mostly selected is the highest resistance and only a high cost of resistance mitigates this behavior. Inversely, we observe that a low resistance strategy can be selected in the auxiliary organ when the development of the tumour is slow and the effect of a large tumour on the mortality of the organism is relatively weak. Nevertheless, if the tumour can spread to a key organ, higher resistance strategies are selected in the auxiliary organ.

**Conclusion:**

Finally, our study demonstrates that the relative contribution of organs to the organism fitness and the metastatic propensity of the tumour influence the evolution of tumour resistance at organ scale and should be considered by studies aiming to explain the variability in cancer incidence at organ-scale.

**Electronic supplementary material:**

The online version of this article (10.1186/s12862-018-1298-7) contains supplementary material, which is available to authorized users.

## Background

**Fig. 1:**
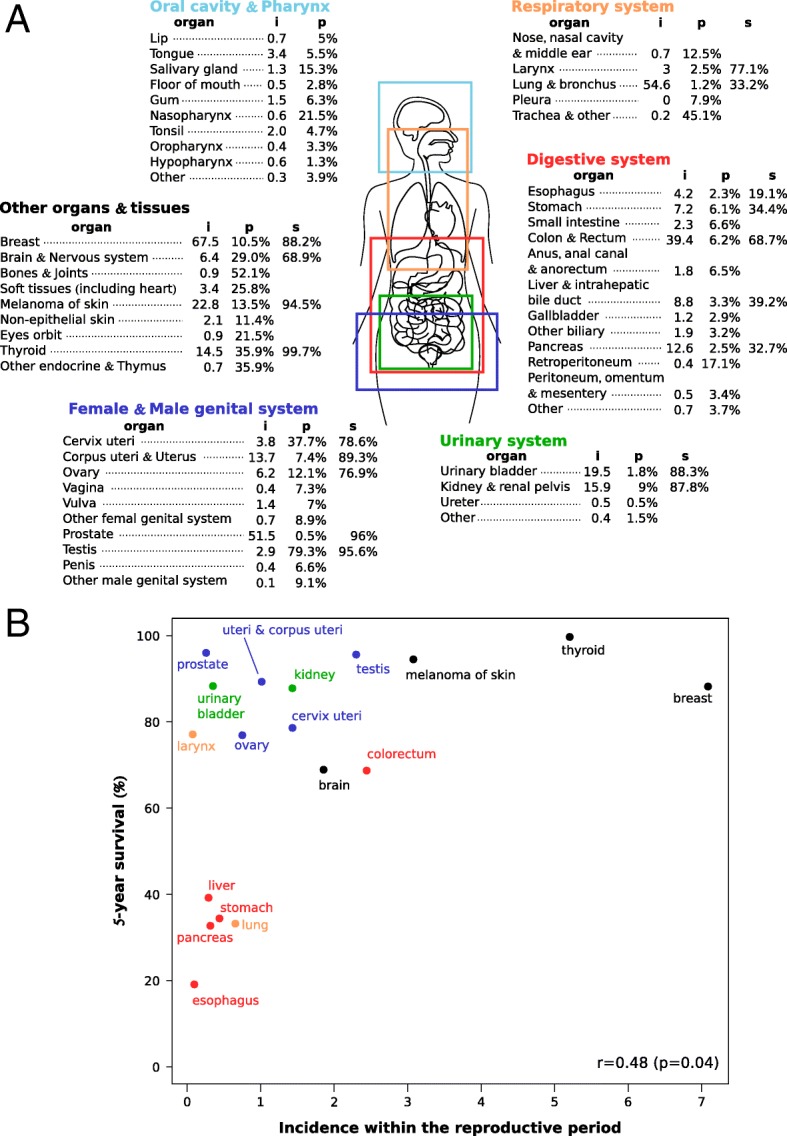
(**a**) The cancer incidence rate (i) and the proportion of incidence cases affecting reproductive people, i.e. age < 45 years, (p) for different organs and tissues. The incidence rate is per 100,000, age adjusted and from individuals without distinction of origin or sex. These data (i and p) correspond to the period 2011–2015. When available, the proportion of cancerous and reproductive people alive 5 years after the diagnosis is reported (s) and correspond to the period 2008–2014. (**b**) Relation between the cancer incidence and the 5-year survival rate for reproductive people, i.e. age < 45 years. Pearson’s correlation test is reported. Data come from the SEER Cancer Statistics Review, 1975–2015, National Cancer Institute [35]⁠

In humans, cancer incidence strongly varies among organs (Fig. 1A). For example, breast cancer is more than 5 times more common than pancreas cancer which is twice more common than brain cancer (Fig. 1A). Moreover, if the pancreas cancer is uncommon among reproductive people (i.e. only 2.5% of pancreas cancers involved people younger than 45 years old), it is also strongly lethal for this population (only 33% survive after 5 years). In contrast, breast cancer is more common among reproductive people (i.e. 10.5% of breast cancers) but is less lethal as 88% survive after 5 years (Fig. 1A).

Various factors have been proposed to explain the variability of cancer incidence at organ-scale such as the different levels of inherited predispositions, carcinogen exposures or stem cell divisions [1–5]⁠. For example, we currently know that the vulnerability of colon to cancer is at least partially due to the high cellular turn-over in this organ and is modulated by the organism’s diet (e.g. obesity, vitamin and calcium deficiency) [6–8]⁠. Interestingly, studies show that the architecture of the colon crypts is able to prevent cancer cell proliferation and is consequently an efficient mechanism against tumour growth [9, 10]⁠. Therefore, the colon has likely evolved a specific tumour suppression mechanism through colon crypts’ architecture to suppress cancer that could prevent individuals to reproduce. More broadly, it seems safe to assume that natural selection acts to decrease cancer risk at organ scale [11–14]. As a support to this assumption, we observe that strongly lethal cancers (such as lung, pancreas, stomach, esophagus and liver cancers for which only 40% of people survive 5 years after the diagnostic) rarely involve reproductive people (Fig. 1B), although some of them are relatively common in the whole population, as the lung cancer (Fig. 1A). In contrast, the most common cancers for reproductive people such as breast cancer, thyroid cancer and melanoma of skin are relatively benigns (Fig. 1B). Overall, it doesn’t exist a strongly lethal cancer affecting people during the reproductive period. Moreover, we observe a positive correlation between cancer incidence within the reproductive period and the survival probability 5 years after the diagnostic (Fig. 1B).

Considering that natural selection acts to decrease cancer risk at organ scale, we can expect that the relative significance of organs to reproduction and survival (until reproduction ceases) have played a crucial role on the evolution of organ-specific tumour resistance mechanisms [15]⁠. If a continuum exists from the most essential organ to organism fitness (e.g. heart, brain, pancreas, sexual organs) to the less essential ones (e.g. gallbladder, adipose tissue), natural selection should be more or less efficient to decrease cancer risk through organ-specific resistance mechanisms depending on the position of the organ in this continuum. Considering only two organs that are distant in this continuum, we expect that even a small tumour in the “key” organ for fitness could lead to a high decrease of organism fitness and that selection acts to decrease cancer risk in this organ through high resistance mechanisms. All things being equal, a tumour (even a large one) in the “auxiliary” organ for fitness, could lead to a smaller decrease of organism fitness and result in lower resistance mechanisms whose development will depend on the associated energetic cost. Finally, metastasis (i.e. the spreading of cancer between organs) could lead to a more complex pattern since even a relatively inoffensive auxiliary-organ tumour can result in a key-organ tumour, and will have after some time a high impact on organism fitness.

The aim of our study is to determine the factors driving the evolution of tumour resistance mechanisms at organ scale when the relative contribution of organs to fitness is considered. We use a population-scale model in which individuals can develop a tumour in a key organ in isolation (scenario 1), in an auxiliary organ in isolation (scenario 2) or in the both organs in interaction through the metastasis (scenario 3). Our simulations show that the mortality rate due to cancer and the mortality rate extrinsic to the cancer are the main drivers of the evolution of tumour resistance mechanisms in the key organ alone and in interaction with the auxiliary organ. Conversely, the evolution of tumour resistance mechanisms in the auxiliary organ strongly depends on its interaction with the key organ. Finally, the relative contribution of organs to the fitness of the organism is likely to have played a crucial role on the current difference of organ vulnerability to cancer and therefore could explain, at least partially, the observed variability of cancer incidence among organs.

## Materials and methods

### The population-scale model

**Fig. 2:**
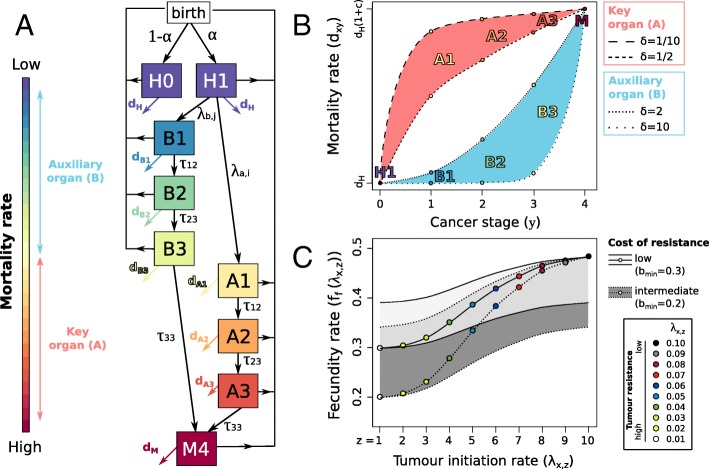
(**a**) A schematic representation of the population-scale model, (**b**) the influence of cancer stage on the mortality rate of individuals for different values of lethality δin the key and the auxiliary organs, and (**c**) the influence of cancer resistance strategies on the fertility rate of individuals for different costs of resistance. In (**b**), the red and the blue area represent the range of mortality rate for individuals with a key-organ cancer and an auxiliary-organ cancer, respectively. In (**c**) the black lines represent the fertility rate of individuals with similar resistance strategies in both organs when they are studied in interaction or in only one organ when they are studied in isolation. The grey areas represent the variability of fertility rates when both organs have different resistance strategies. Solid black line and light grey area correspond to a low cost of resistance and dashed black line and dark grey area correspond to an intermediate cost of resistance. For the sake of legibility, the high cost of resistance (*b*_*min*_ = 0.1) is not represented

We model a logistically growing population of individuals that are classified according to their cancer status as follows (Fig. 2A):

1 $$ {\displaystyle \begin{array}{c}\frac{dH0}{dt}=\left(1-\upalpha \right)\ast {f}_f\left({\uplambda}_a,{\uplambda}_b\right)\ast N\ast \left(1-\left(N/K\right)\right)-{d}_HH0+{f}_m(H0)\\ {}\frac{dH1}{dt}=\upalpha \ast {f}_f\left({\uplambda}_a,{\uplambda}_b\right)\ast N\ast \left(1-\left(N/K\right)\right)-\left({\uplambda}_a+{\uplambda}_b+{d}_H\right)H1+{f}_m(H1)\\ {}\frac{dA1}{dt}={\uplambda}_aH1-\left({\uptau}_{12}+{d}_{A1}\right)A1+{f}_m(A1)\\ {}\frac{dB1}{dt}={\uplambda}_bH1-\left({\uptau}_{12}+{d}_{B1}\right)B1+{f}_m(B1)\\ {}\frac{dA2}{dt}={\uptau}_{12}A1-\left({\uptau}_{23}+{d}_{A2}\right)A2+{f}_m(A2)\\ {}\frac{dB2}{dt}={\uptau}_{12}B1-\left({\uptau}_{23}+{d}_{B2}\right)B2+{f}_m(B2)\\ {}\frac{dA3}{dt}={\uptau}_{23}A2-\left({\uptau}_{33}+{d}_{A3}\right)A3+{f}_m(A3)\\ {}\frac{dB3}{dt}={\uptau}_{23}B2-\left({\uptau}_{33}+{d}_{B3}\right)B3+{f}_m(B3)\\ {}\frac{dM}{dt}={\uptau}_{33}A3+{\uptau}_{33}B3-{d}_MM+{f}_m(M)\end{array}} $$

, where *N* and *K* are the total number of individuals in the population and the carrying capacity respectively. *H*represents healthy individuals, *A *and *B* correspond to individuals with a tumour in a key and an auxiliary organ, respectively. Among healthy individuals, we distinguish those who will never develop a tumour (*H*0) and individuals who are developing a tumour (*H*1). The proportion of individuals in each group is shaped by the parameter αwhich represents the rate of tumour emergence in the population and allow to consider both the concomitant influence of genetic susceptibility and environmental exposure to carcinogens. Among individuals with a tumour in a key or an auxiliary organ, we distinguish four tumour stages: tumour initiation (e.g. carcinoma in situ, stage 1), large tumour invading the major part of the organ (e.g. invasive tumour, stage 2), tumour that can migrate to other distant organs (e.g. possible metastatic cancer, stage 3) and the metastatic stage (stage 4) which corresponds to individuals with a tumour in both the key and the auxiliary organs (*M*). Healthy individuals*H*1can develop a tumour of stage 1 in a key organ (*A*1) or an auxiliary organ (*B*1) through the tumour initiation rate λ_*a*_ and λ_*b*_, respectively. Individuals with a tumour of stage 1 (*A*1 and *B*1) or stage 2 (*A*2 and *B*2) can develop a tumour of stage 2 or stage 3 through the rate τ_12_ and τ_23_, respectively. Finally, individuals with a tumour of stage 3 (*A*3 and *B*3) can develop a metastatic cancer (*M*) through the rate τ_33_. We assume that tumour resistance mechanisms impact only the initiation rates in the key and auxiliary organs (λ_*a*_ and λ_*b*_, respectively), where low values of tumour initiation rates represent high resistance mechanisms.

Individuals give birth at a fecundity rate that depend on the tumour resistance mechanisms (*f*_*f*_(λ_*a*_, λ_*b*_)). In other words, we consider a resistance-fecundity trade-off for which high tumour resistance mechanisms have a cost on organism fitness through a reduction in fecundity rate (Fig. 2C and Additional file 1 for more details).

We also consider two different kind of mortality rates: the extrinsic mortality rate (*d*_*H*_) corresponding to all sources of death that are not directly linked to cancer (e.g. predation, parasitism, harsh environmental conditions) and the related-cancer mortality rate (*d*_*xy*_) which depends on the organ affected*x*(i.e. the key organ, A, the auxiliary organ, B, neither of the two, H, or both, M) and the tumour stage*y* (1, 2 or 3) (Fig. 2B). We assume that the mortality of individuals with a metastatic cancer (*M*) increases by a factor of (1 + *c*) the mortality of individuals:

$$ {d}_M={d}_H\left(1+c\right) $$

where *c* is a constant to manage the strength of cancer impact on mortality and varies from 1 to 19 (Table 1).

**Table 1:** Description of the main parameters and their values. The changes in parameter values because of the scenario studied are described for the key organ in isolation (scenario 1), the auxiliary organ in isolation (scenario 2) and the both organs in interaction (scenario 3). The last five lines correspond to the parameters used in the sensitivity analysis

Parameter	Description	Range or value
α	Tumour emergence rate	0.5, 0.75, 1.
λ_*a*, *i*_	Cancer initiation rate in the key organ	If scenario 1 or 3, λ_*a*, *i*_ = 0.01 ∗ *i* If scenario 2, λ_*a*_ = 0
λ_*b*, *j*_	Cancer initiation rate in the auxiliary organ	If scenario 2 or 3, λ_*b*, *j*_ = 0.01 ∗ *j* If scenario 1, λ_*b*_ = 0
*i* and *j*	Cancer resistance strategy in the key and the auxiliary organ, respectively.	*ℕ* ∈ [1, 10]
*b*_*min*_	The minimal and maximal fecundity rates	0.1, 0.2, 0.3
*b*_*max*_		0.5
*K*	Carrying capacity	10,000
*d*_*H*_	Mortality rate of healthy individuals	*d*_*H*_ ∼ *Unif*(10^−2^, 5.10^−2^)
*m*	Mutation rate	*m* ∼ *Unif*(10^−10^, 10^−6^)
τ_12_, τ_23_ and τ_33_	Probability to have a cancer of stage 2, 3 and 4 (metastasis) when you have a cancer of stage 1, 2 and 3, respectively.	τ_12_ ∼ *Unif*(0.1, 0.9) τ_23_ ∼ *Unif*(0.1, 0.9) If Scenario 3, τ_33_ ∼ *Unif*(0.1,0.9) If Scenario 1 or 2, τ_33_ = 0
δ	The lethality of cancer in the key and the auxiliary organ	δ ∼ *Unif*(1/10, 1/2)for the key organ and δ ∼ *Unif*(2, 10) for the auxiliary organ
*c*	Constant to manage the strength of cancer impact on organism’s mortality	*c* ∼ *Unif*(1, 19)

An important feature of our model is that even a small tumour (stage 1) in a key organ (*A*) increases significantly and immediately the mortality of the organism whereas only a large tumour (stage 3) in an auxiliary organ (*B*) has a relatively high impact on the mortality of the organism (Fig. 2B). This specific behavior is modeled using an unique parameter δ whose the value depends on the organ studies (i.e. δ < 1 for the key organ and δ > 1 for the auxiliary organ) and which manages the shape of the relationship between cancer development and the cancer-related mortality rate (Fig. 2B, Additional file 1).

Finally, we extend the model to permit that the population is composed by individuals with different tumour resistance strategies represented by different values of cancer initiation rate λ_*a*_ or λ_*b*_ in scenario 1 and 2 respectively, and different combinations of cancer initiation rates λ_*a*_ and λ_*b*_ in scenario 3. We use the subscripts *i*and *j*to identify the resistance strategies of the key and the auxiliary organs respectively, λ_*a*, *i*_ and λ_*b*, *j*_. The evolution of these strategies requires that an individual transmits its resistance strategy to its descendants, but also that a mutation event can change the strategy of an individual. The mutation function *f*_*m*_ permits the evolution of these different tumour resistance strategies at a mutation rate *m* (Additional file 1).

### Numerical simulations

We use numerical simulations to determine the level of tumour resistance selected in a key organ and in an auxiliary organ for the different scenarios studied. For each organ, we explore 10 different levels of tumour resistance, corresponding to 100 possible strategies when the organs are in interaction (100 different pairs of λ_*a*, *i*_ and λ_*b*, *j*_ in the scenario 3). The initial population contains 10^4^ healthy individuals (*H*0 and *H*1) and the different strategies are equally represented among these individuals. We let the population evolve until reaching a stable equilibrium (11,000 years have been used). Finally, we consider as the selected resistance mechanism in the key organ (λ_*a*_) or in the auxiliary organ (λ_*b*_), the resistance mechanism which is the most frequent in those organs within the population at the end of the simulation.

### Sensitivity analysis

We test the effect of all parameters and functions on our results. We evaluate the effects of tumour emergence variability (α) and the effect of the shape of the fecundity-resistance trade-off (*f*_*f*_) in the Additional file 2. Concerning the parameters for which we want to itemize the impact on resistance strategies at organ-scale (the parameters in the last five lines of the Table 1), we explore a large range of values using a sensitivity analysis.

The parameters used in the sensitivity analysis describe either the population, the organ or the tumour properties. First, the mutation rate (*m*), the cancer-related organism mortality rate (*c*) and the extrinsic organism mortality rate (*d*_*H*_) are population properties describing the stability of evolutionary strategies in the population and its vulnerability to cancer and all other sources of death. Because the mutation rate never influences the evolution of resistance mechanisms in organs, we no longer discuss on it in this paper. Second, the lethality of tumour stages (δ) is an organ property allowing the distinction between a key organ (δ < 1) and an auxiliary organ (δ > 1) (Fig. 2B). Moreover, the variability of δ allows to vary the effect of a small tumour in the key organ and the effect of large tumour in the auxiliary organ for organism’s mortality (Fig. 2B). Third, the speed of tumour development (τ_12_, τ_23_and τ_33_) is a tumour property.

To evaluate the uncertainty inherent to the variability of parameters, we perform a sensitivity analysis using a Latin Hypercube Sampling (LHS) approach provided by the ‘lhs’ package in R 3.4.0. We generate 200 sets of the parameter values through the LHS algorithm, where each parameter varies uniformly from 67 to 100% around its average value, depending on the parameter (Table 1). Then, we explore the 200 parameter combinations to evaluate the uncertainty in parameter values. For each scenario (i.e. key or auxiliary organs in isolation or in interaction), we perform 200 simulations corresponding to the 200 parameter combinations from the sensitivity analysis.

We investigate the relative contribution of the seven parameters to the variability of the selected resistance strategies (λ_*a*_ and λ_*b*_) by calculating the Partial Rank Correlation Coefficient (PRCC). The PRCC provides a measure of the strength of the monotonic non parametric correlation between a parameter and the selected resistance strategy, after the linear effects of the remaining parameters have been considered. The strength of the correlation is represented by the absolute value of the PRCC and the direction of the correlation by the sign of the PRCC. By combining the LHS with PRCC, we are able to reasonably assess the sensitivity of our model outcomes to the variability of initial parameters [16]⁠.

## Result

### Selected resistance mechanisms

**Fig. 3:**
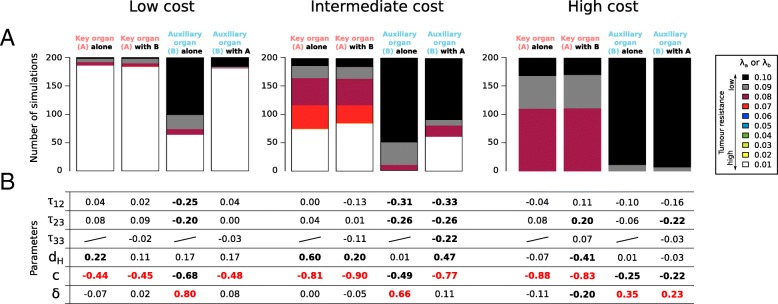
**a**) The proportion of the different selected resistance mechanisms and **b**) their sensitivity to initial parameters for a low, an intermediate and a high cost of resistance (*b*_*min*_ = 0.3, *b*_*min*_ = 0.2 and *b*_*min*_ = 0.1 respectively) for the key organ and the auxiliary organ considered in isolation and considered in interaction through metastasis. In **b**) the Partial Rank Correlation Coefficient (PRCC) are calculated between the seven input parameters and the selected resistance mechanisms in the key organ (λ_*a*_) and the auxiliary organ (λ_*b*_). The significant correlations are in bold (t-test, *p* < 0.01). The main selective forces are in red

When the organs are considered in isolation (scenario 1 and 2), our results reveal a clear difference between tumour resistance strategies in the key and the auxiliary organs. For a relatively low cost of resistance mechanisms (*b*_*min*_ = 0.3), the strongest resistance strategy is mainly selected in the key organ whereas a large diversity of resistance strategies is observed in the auxiliary organ (Fig. 3A). When the resistance to tumour has an intermediate cost for the organism (*b*_*min*_ = 0.2), we observe a higher diversity of selected resistance strategies in the key organ whereas the weakest resistance strategy is mainly selected in the auxiliary organ (Fig. 3A). As expected, a higher cost of cancer resistance (*b*_*min*_ = 0.1) leads to weaker resistance strategies selected in the key and the auxiliary organs. In particular, we never observe the highest resistance strategy, even in the key organ, and we observe a very low diversity of resistance strategy selected in the auxiliary organ as the weakest resistance strategy is selected in more than 94% of cases in this organ (Fig. 3A).

The selected strategies in the key organ are mostly the same if it is considered alone or in interaction with the auxiliary organ (Figs. 3A). In contrast, the selected strategies in the auxiliary organ become more resistant to tumour when the organs are in interaction. Interestingly, when the cost of resistance is low (*b*_*min*_ = 0.3), the selected resistance mechanisms in the auxiliary organ studied in interaction with the key organ (scenario 3) are approximately similar to those selected in the key organ (Fig. 3A). When the cost of resistance is high (*b*_*min*_ = 0.1), they are similar to those selected in the auxiliary organ studied in isolation (Fig. 3A). And finally, when the cost of resistance is intermediate (*b*_*min*_ = 0.2), they are a compromise between the selected resistance in the auxiliary organ studied in isolation and in the key organ (Fig. 3A). These results suggest that the cost of resistance strongly influences the selection of mechanisms in the auxiliary organ and thus the timing of cancer spread between organs.

### The factors influencing the evolution of tumour resistance mechanisms

We analyse here the effect of parameters on the resistance mechanisms selected in the key and the auxiliary organs considered in isolation (scenario 1 and 2). The effect of interactions between these organs through metastasis (scenario 3) is discussed later.

### Organs considered in isolation (scenario 1 and 2)

**The cancer-related mortality rate (***c***)** is negatively correlated to the selected tumour initiation rates λ_*a*_and λ_*b*_in both the key and the auxiliary organs and whatever the cost of the resistance (Fig. 3B), meaning that an increase of mortality due to cancer will be associated with the selection of stronger resistance. In the key organ, the cancer-related mortality rate is the main selective force of resistance mechanisms (Fig. 3B).

**The extrinsic mortality rate (***d*_*H*_**)** also influences the selected resistance mechanism in the key organ (λ_*a*_) but at a lower level than the cancer-related mortality rate (*c*) (Fig. 3B). The correlation between *d*_*H*_ and λ_*a*_ is positive which means that weaker resistance mechanisms are selected in the key organ when the extrinsic mortality rate is higher. The extrinsic mortality rate has no influence on the selected resistance in the auxiliary organ (Fig. 3B).

**The lethality of tumour stages (**δ**)** never influences the selected resistance strategy in the key organ mainly because the variability of δ leads to a relatively low variability of the mortality of individuals with a three-stage key organ tumour (*A*3) (Fig. 2B). In other words, whatever the value of the lethality of tumour stages is, the key organ tumour leads to a high mortality rate of the organism at the end. Whatever the cost of tumour resistance for the individual, the variability of the lethality of tumour stages doesn’t lead to a strong variation of selection pressure in the key organ and thus doesn’t drive a change on tumour resistance strategies. On the other hand, δ has a stronger impact on the selected resistance mechanisms of the auxiliary organ than the cancer-related mortality rate (*c*) (Fig. 3B). High δ values lead to a relatively low mortality of individuals with a three-stage auxiliary organ tumour (i.e. closed to the mortality of healthy individuals, Fig. 2B). Inversely, low δ values lead to a high mortality of individuals with a three-stage auxiliary organ tumour (i.e. closed to the mortality of a first-stage key organ tumour, Fig. 2B). Consequently, δ strongly drives the impact of the auxiliary organ tumour on mortality and the efficiency of auxiliary tumour resistance strategies are selected based on this parameter.

**The speed of tumour progression (**τ_12_ **and** τ_23_**)** is negatively correlated with the tumour initiation rates selected in the auxiliary organ (λ_*b*_), except when the cost of tumour resistance is high (Fig. 3B). This correlation means that strong resistance mechanisms are selected to prevent a rapidly progressing tumour in this organ. In the auxiliary organ, only a third-stage tumour may result in a relatively high mortality rate. Consequently, there is a likely strong advantage in delaying the progression of tumour in this organ to delay its impact on organism fitness. Inversely, we observe no effect of tumour progression speed on resistance selected in the key organ. A tumour in the key organ leads to an immediately high mortality of the organism after its initiation and thus the benefit of delaying the progression of this tumour is probably too small in comparison with the cost associated to the resistance.

### Organs in interaction via metastases (scenario 3)

When both organs are in interaction, a tumour in an auxiliary organ could result at the end in a tumour in a key organ (i.e. metastasis). One of the consequences is that the selection favours the evolution of more efficient resistance mechanisms in the auxiliary organ interacting with the key organ compared with the auxiliary organ considered in isolation, except when the cost of tumour resistance is high (Fig. 3A). Another consequence is that the main selective force influencing the resistance mechanisms in the auxiliary organ is not the same when this organ is considered in isolation or in interaction with the key organ (Fig. 3B). Whereas the lethality of tumour stages (δ) is the main driver in the auxiliary organ considered in isolation (leading to a tolerance strategy when the effect of tumour on fitness is relatively weak), this parameter has no influence on the selected resistance mechanisms in the auxiliary organ in interaction with the key organ (Fig. 3B). We observe an exception to this behavior when the cost of tumour resistance is high because in this condition, the weakest tumour resistance strategy is selected in the auxiliary organ in 94% of simulations (Fig. 3A). In interaction with the key organ, the main driver of tumour resistance evolution in the auxiliary organ is the cancer-related mortality rate (*c*) which is also the main driver of the key organ alone or in interaction (Fig. 3B). Interestingly, high resistance mechanisms are also selected in the auxiliary organ in interaction with the key organ to prevent rapidly progressing tumours (high τ_12_and τ_23_) and rapidly spreading cancers (high τ_33_) when the cost of resistance is high enough to prevent the evolution up to the highest tumour resistance for all parameter combinations (low cost,*b*_*min*_ = 0.3, compared with intermediate cost, *b*_*min*_ = 0.2, Fig. 3). In this condition, there is a likely strong advantage in delaying the progression and spreading of cancer in the auxiliary organ to delay the strong impact it can have on fitness by affecting the key organ.

## Discussion

If the risk of developing cancer increases with age, it remains clear that most cancers may appear during the reproductive period (Fig. 1) [17–19]⁠. Consequently, the cancer has been and is still a selective pressure for most of multicellular organisms and natural selection has shaped various protective mechanisms to prevent or delay the effect of cancer on fitness [11–14, 20]⁠. In line with this idea, an important feature of our model is that the reproduction occurs throughout the life of individuals. This amounts to consider cancer no longer as a disease affecting mainly the elderly but as a set of oncogenic events (from carcinoma in situ to metastatic cancer) initiated during the reproductive period. In our model, a tumour may have a large diversity of effects on fitness depending on the relative contribution of the affected organ to the individual’s reproduction and survival (Fig. 2B), from strong and rapid effects on fitness for key-organ tumours to weak and slow effects for auxiliary-organ tumours.

The objective of this paper is to determine and discuss the factors driving the evolution of tumour resistance mechanisms at organ-scale when the relative contribution of organs to the organism fitness is taken into account. Our simulations show that natural selection acts in two different ways to prevent cancer in a key and an auxiliary organs: the strategy mostly selected in the key organ is the highest resistance whereas a low resistance strategy can be selected in the auxiliary organ when the development of the tumour is slow and the effect of a large tumour on the mortality of the organism is relatively weak (Fig. 3). In our model, we assume that reproductive success of individuals accumulates through time and the development of tumour resistance mechanisms has a cost for the organism through a decrease of fecundity. Consequently, the lower fecundity rate of individuals with high tumour resistance mechanisms have to be compensated by a significantly longer lifespan to reach a similar fitness compared to their highly fecund and tumour prone competitors. By definition, a key organ tumour has a higher negative impact on organism’s lifespan than an auxiliary organ tumour (Fig. 2B), thus a resistance mechanism leads to a higher advantage when it protects a key organ compared to an auxiliary organ. This explains why, if all else is equal, natural selection should favour higher tumour resistance mechanisms in a key organ compared with an auxiliary organ (Fig. 3). However, the propensity of the tumour to spread between organs modulates this result. Indeed a high metastatic propensity of the tumour (i.e. large τ_33_) leads to higher resistance strategies in the auxiliary organ to prevent the spread of tumour to the key organ (Fig. 3).

In this study, the challenge is to develop the most parsimonious model able to integrate important and complex processes and bring insight on the evolution of tumour resistance at organ-scale. To do that, we consider two important assumptions that we discuss here because they have a strong influence on our results.

First, we assume that tumour resistance mechanisms have a cost for the organism through a decrease of fecundity and our results show that more this cost is high and more the tumour resistance mechanism selected at organ scale is weak. Theoretical studies and clinical evidences showed that evolutionary trade-offs involving tumour resistance mechanisms and reproductive abilities exist. For example, studies have shown that specific mutations in genes associated with cancer defences (e.g. genes from the cancer suppressor or from the deoxyribose nucleic acid repair family) may be simultaneously involved with higher susceptibility to cancer and also with increased reproductive potentials [21, 22]. In line with previous studies, our modelling approach shows that a trade-off between fecundity and tumour resistance can play a crucial role in the evolutionary trajectories of resistance to tumour [23, 24]⁠. For example, the highest tumour resistance mechanism is mainly selected in the key organ when the cost of resistance is low whereas it is never selected when the cost is high (Fig. 3). However, if a trade-off between tumour resistance and fecundity seems to be a reasonable way to model the cost of tumour resistance mechanisms, it is probably not the only one involving tumour resistance. For example, other trade-offs between cancer and immune response to other diseases and pathogens [25]⁠ or tissue repair mechanisms [26]⁠ are suggested so far and many more are probably still to discover. Moreover, an evolutionary process called antagonistic pleiotropy suggests in the context of cancer that pleiotropic genes could have both a positive effect on fitness and tumour development [26–28]⁠.

Second, our results reveal that the efficiency of tumour resistance selected in auxiliary organs may strongly depend on the ability of cancer cells to spread and establish in key organs. In this study, we use a model with two organs which represents the simplest organ network allowing us to study the potential effect of metastases on organ resistance. Considering that the human body is constituted by numerous organs and tissues connected by a complex network of interactions, it appears as an interesting perspective for our work to take into account a larger number of organs, although it would significantly complexify the model (one organ is represented by three equations in the equation system 1). Moreover, the spreading of cancer in our organ network occurs only after the primary tumour has reached the last stage of development (i.e. the third stage). This is a necessary assumption because the modalities of cancer cell spreading in the body are still unknown. Especially it is unclear if cancer cells start spreading with the formation of premalignant lesions, or directly after the primary tumour appears or after the time necessary for the tumour growth to be constraint by the initial environment [29, 30]. However, our model allows to easily test an alternative hypothesis such as the spread of cancer cells directly after the initiation of the primary tumour. On the other hand, what is clear is that certain initial tumour in a specific organ preferentially spread in a set of metastatic organs, even if the mechanisms are still debate [29–33]. A very interesting perspective for our model would be to consider explicitly these pathways.

An important aspect of our model is that the evolution of tumour resistance mechanisms is modeled at the population level, allowing us to study the effect of different levels of extrinsic mortality rate (*d*_*H*_) on the evolution tumour resistance at organ-scale. In our model, the more the extrinsic mortality rate increases and the less the cancer is a likely cause of deaths in the population. Therefore, the variation of the extrinsic mortality rate may represent the variation of the harshness of environmental conditions for our population: a high *d*_*H*_ corresponding to a wild population dealing with harsh conditions (e.g. a high level of predation, a low level of resource availability) and a low *d*_*H*_ corresponding to a population enjoying more comfortable conditions in a safer environment (e.g. a low level of predation, a high level of resource availability). Interestingly, we observe a positive correlation between *d*_*H*_ and the tumour initiation rate of the key and the auxiliary organs (λ_*a*_ and λ_*b*_, respectively) which suggests that populations dealing with harsh conditions invest less in tumour resistance mechanisms at organ scale than populations in safer conditions. However, this correlation can also be shaped by the allometric constraint linking fecundity and tumour resistance in our model. The increase of the extrinsic mortality may result in an increase of the selective advantage of individuals with a high fecundity rate which leads to the spread of weak resistance. Even if our model doesn’t allow disentangling the causes-consequences of the co-variations between mortality-fecundity and mortality-resistance, we show that tumour resistance mechanisms at organ-scale are strongly influenced by extrinsic mortality of the population when those mechanisms have a reproductive cost for the organism. This finding suggests that, within the same species, resistance mechanisms selected at organ-scale could differ between wild populations and populations having evolved in safe conditions (such as zoo or protected area). Moreover, our model doesn’t consider that cancer is likely to be an important indirect cause of deaths in wild populations where even a small decrease of body conditions could result in a strong reduction of abilities to find resources or to escape predators, parasites or infections [20, 34]⁠. Consequently, the impact of cancer on mortality of individuals is likely underestimated in our model for wild populations (i.e. high value of *d*_*H*_) and could be more fairly estimated by adding a positive interaction between the extrinsic mortality rate (*d*_*H*_) and the cancer-related mortality rate (*c*).

## Conclusion

Our study provides the first example of how the different contributions of organs to the individual’s reproduction and survival may have influenced the evolution of tumour resistance mechanisms at organ-scale. This is a first step to better understand the variability of cancer incidence among organs using evolutionary theories. How realistic our predictions are? For most species, it is currently impossible to provide an exact response due to an obvious lack of data. For humans, we have some promising data sets [35], but given the diversity and complexity of factors that can influence cancer incidence (e.g. inherited predisposition, carcinogen exposure, number of stem cell division) the analysis of such data to test our predictions is likely challenging and could represent an intriguing avenue for new research.

## Additional files

Additional file 1: Supplementary methods. This file contains the description of 1) the extended model for different tumour resistance strategies, 2) the mutation function, 3) the fecundity function, 4) the mortality function and a summary of all the functions used in the article (Table S1). (ODT 198 kb)

Additional file 2: Supplementary results. This file contains a description of the effect of tumour emergence variability on the tumour resistance mechanisms selected at organ-scale. It also contains a test to determine the influence of using Gompertz function to model the trade-off between tumour initiation rate and fecundity rate in the outcomes. (ODT 1764 kb)

## Abbreviations

ANR: Agence Nationale de la Recherche
DNA: Deoxyribose Nucleic Acid
EVOCAN: EVOlution of CANcer
LHS: Latin Hypercube Sampling
NCI: National Cancer Institute
PRCC: Partial Rank Correlation Coefficient
SEER: Surveillance, Epidemiology, and End Results

## References

[1] Ashford NA, Bauman P, Brown HS, Clapp RW, Finkel AM, Gee D (2015). Cancer risk : role of environment. Science (80- )..

[2] Song M, Giovannucci EL (2015). Cancer risk: Many factors contribute. Science (80- )..

[3] Tomasetti C, Vogelstein B (2015). Variation in cancer risk among tissues can be explained by the number of stem cell divisions. Science (80- ).

[4] Wild C, Brennan P, Plummer M, Bray F, Straif K, Zavadil J (2015). Cancer risk: role of chance overstated. Science (80- )..

[5] Wu S, Powers S, Zhu W, Hannun YA (2015). Substantial contribution of extrinsic risk factors to cancer development. Nature.

[6] Moghaddam AA, Woodward M, Huxley R (2007). Obesity and risk of colorectal cancer: a meta-analysis of 31 studies with 70,000 events. Cancer Epidemiol Biomark Prev.

[7] Ryan-Harshman M, Aldoori W (2007). Diet and colorectal cancer: review of the evidence. Can Fam Physician.

[8] Ma Y, Yang Y, Wang F, Zhang P, Shi C, Zou Y (2013). Obesity and risk of colorectal Cancer: a systematic review of prospective studies. PLoS One.

[9] Michor F, Frank SA, May RM, Iwasa Y, Nowak MA (2003). Somatic selection for and against cancer. J Theor Biol.

[10] Gatenby RA, Gillies RJ, Brown JS (2010). Evolutionary dynamics of cancer prevention. Nat Rev Cancer.

[11] Leroi AM, Koufopanou V, Burt A (2003). Cancer selection. Nat Rev Cancer.

[12] Cairns J (1975). Mutation selection and the natural history of cancer. Nature.

[13] Caulin AF, Maley CC (2011). Peto’s paradox: Evolution’s prescription for cancer prevention. Trends Ecol Evol.

[14] Crespi B, Summers K (2005). Evolutionary biology of cancer. Trends Ecol Evol.

[15] Thomas F, Nesse RM, Gatenby R, Gidoin C, Renaud F, Roche B (2016). Evolutionary ecology of organs: a missing link in Cancer development?. Trends in Cancer.

[16] Marino S, Hogue IB, Ray CJ, Kirschner DE (2008). A methodology for performing global uncertainty and sensitivity analysis in systems biology. J Theor Biol.

[17] World Health Organization. Mortality, Database. http://www.who.int/healthinfo/mortality_data/en/.

[18] Hueper AWC (1952). Age aspects of environmental and occupational cancers. Public Health Rep.

[19] Bleyer A, Barr R, Hayes-Lattin B, Thomas D, Ellis C, Anderson B (2008). The distinctive biology of cancer in adolescents and young adults. Nat Rev Cancer.

[20] Vittecoq M, Roche B, Daoust SP, Ducasse H, Missé D, Abadie J (2013). Cancer: a missing link in ecosystem functioning?. Trends Ecol Evol.

[21] Kang H-J, Feng Z, Sun Y, Atwal G, Murphy ME, Rebbeck TR (2009). Single-nucleotide polymorphisms in the p53 pathway regulate fertility in humans. Proc Natl Acad Sci U S A.

[22] Smith KR, Hanson HA, Mineau GP, Buys SS (2012). Effects of BRCA1 and BRCA2 mutations on female fertility. Proc R Soc B Biol Sci.

[23] Boddy AM, Kokko H, Breden F, Wilkinson GS, Aktipis CA. Cancer susceptibility and reproductive trade-offs: a model of the evolution of cancer defences. Philos Trans R Soc B Biol Sci. 2015;370. 10.1098/rstb.2014.0220. 10.1098/rstb.2014.0220 PMC4581025 26056364

[24] Jacqueline C, Biro PA, Beckmann C, Moller AP, Renaud F, Sorci G (2017). Cancer: a disease at the crossroads of trade-offs. Evol Appl.

[25] Ukraintseva SV, Arbeev KG, Akushevich I, Kulminski A, Arbeeva L, Culminskaya I (2010). Trade-offs between cancer and other diseases: do they exist and influence longevity?. Rejuvenation Res.

[26] Weinstein BS, Ciszek D (2002). The reserve-capacity hypothesis: evolutionary origins and modern implications of the trade-off between tumor-suppression and tissue-repair. Exp Gerontol.

[27] Carter AJR, Nguyen AQ (2011). Antagonistic pleiotropy as a widespread mechanism for the maintenance of polymorphic disease alleles. BMC Med Genet.

[28] Ungewitter E, Scrable H (2009). Antagonistic pleiotropy and p53. Mech Ageing Dev.

[29] Sahai E (2007). Illuminating the metastatic process. Nat Rev Cancer.

[30] Valastyan S, Weinberg R (2011). Tumor metastasis: molecular ınsights and evolving paradigms. Cell.

[31] Paget S (1889). Distribution of secondary growths in cancer of the breast. Lancet.

[32] Paterlini-Bréchot P (2014). About seed and soil. Cancer Microenviron.

[33] Scott J, Kuhn P, Anderson ARA (2012). Unifying metastasis — integrating intravasation, circulation and end-organ colonization. Nat Rev Cancer.

[34] Vittecoq M, Ducasse H, Arnal A, Møller AP, Ujvari B, Jacqueline CB (2015). Animal behaviour and cancer. Anim Behav.

[35] Noone A, Howlader N, Krapcho M, Miller D, Brest A, Yu M, et al. SEER Cancer Statistics Review, 1975–2015, National Cancer Institute. Bethesda, MD, based on November 2017 SEER data submission, posted to the SEER web site, April 2018. https://seer.cancer.gov/csr/1975_2015/.

