# Supplementary material for: How is the evolution of tumour resistance at organ-scale impacted by the importance of the organ for fitness?
Source: BMC Evol Biol. 2018 Dec 6;18:185. doi: 10.1186/s12862-018-1298-7 (PMC6282255; doi:10.1186/s12862-018-1298-7)
Supplement: Supplementary file 1 [file 12862_2018_1298_MOESM1_ESM.odt]

# Supplementary methods

## The extended model for different tumour resistance strategies

For simplicity, we have only described in the main text the model with a single tumour resistance strategy that is described by a single combination of  and . Here, we present the extended model describing the population composed by individuals with different tumour resistance strategies and we use the subscripts  and  to identify the strategies of the key and the auxiliary organs respectively.

|  | 1 |
| --- | --- |

## The mutation function

The evolution of strategies requires that an individual transmits its resistance strategy to its offsprings, but also that a mutation event can change the strategy of an individual. We assume that mutations altering resistance strategies are gradual and not punctuated. Consequently, a mutation leads to either a slightly weaker resistance strategy for one organ (e.g. a resistance strategy evolving from  to , with <) or a slightly higher resistance strategy (e.g. a resistance strategy evolving from  to   with ). We model this process using a mutation rate () and a mutation function () upon individual’s birth which gradually increases or decreases the tumour initiation rates by small increments:

| formula | 2 |
| --- | --- |

A mutation at the boundaries of resistance strategies can only lead to a weaker (higher) resistance strategy for individuals at the upper (lower) boundary of resistance strategies. For example, the individuals with the weakest and the highest resistance strategies in the key and the auxiliary organ respectively () can only evolve to a higher resistance strategy in the key organ () and a weaker resistance strategy in the auxiliary organ (). We get: 

| formula | 3 |
| --- | --- |

## The fecundity function

We assume that high resistance mechanisms have a cost through a reduction in fecundity rate. In other words, the increase of tumour initiation rates ( or ) leads to an increase in the fecundity rate of the individual, . First, we assume that the fecundity-resistance trade-off is modelled through a Gompertz function (this hypothesis is relaxed in Supplementary Results): 

|  | 4 |
| --- | --- |

,where  and  correspond to the fecundity rate of individuals with tumour initiation rates  and  for the key and the auxiliary organ respectively, and  are the minimal and maximal fecundity rates, and  are two constants affecting the shape of the trade-off. 

We vary the cost of tumour resistance using the parameter  ( is fixed and equal to 0.5 throughout the study). We consider that the cost of resistance is relatively low when  , intermediate when formule and relatively high when  (Figure 1C). We also consider that the range of fecundity rates () varies according to the cost of resistance but is independent on the number of studied organs (Figure 1C). Finally, to make the comparison between the key and the auxiliary organ in isolation (Scenario 1 and 2) and the both organs in interaction (Scenario 3) easier, we assume that the fecundity rate of an individual () depends equally on the resistance strategies of both organs when they are in interaction and exclusively on the resistance strategy of the studied organ when it is in isolation (Table S1). This hypothesis is relaxed in Supplementary Results.

## The mortality function

In our model, the tumour has an impact on fitness by increasing the mortality of its host. We assume that the mortality of individuals with a metastatic cancer (stage 4) increases by a factor of () the mortality of individuals:, where  is a constant to manage the strength of cancer impact on mortality and varies from 1 to 19 (Table 1). Concerning non-metastatic cancers, the effect of a tumour on the mortality () depends on the organ affected  (i.e. the key organ A or the auxiliary organ B), the tumour stage  (1, 2 or 3) and respects the following condition  (Figure 1B). We choose:

|  | 5 |
| --- | --- |

where is a parameter to manage the lethality of tumour stages and whose the value depends on the organ affected.

For the key organ,which ensures that a small tumour (stage 1) increases significantly the mortality of its host, and for the auxiliary organ,  which ensures that only a large tumour (stage 3) has a relatively high impact on the mortality of its host. 

## A summary of the functions

Table S1: The description of the functions used in the right part of the equation 1 in the order they appear. The changes in functions because of the scenario studied are described for the key organ studied in isolation (scenario 1), the auxiliary organ studied in isolation (scenario 2) and the both organs studied in interaction (scenario 3). 

| Function | Description | Equation |
| --- | --- | --- |
|  | Fecundity function | If scenario 1,   If scenario 2, If scenario 3, With, where and . |
|  | Number of individuals with a tumour resistance strategy  in the key organ and  in the auxiliary organ. |  |
|  | The total number of individuals in the population |  |
|  | Mutation function | With |
|  | Mortality function | With  corresponding to the organ affected (i.e. the key organ A or the auxiliary organ B)  corresponding to the tumour stage (1, 2, 3). When the tumour stage is maximal (), both organs are affected and we get: |
