# Supplementary material for: How is the evolution of tumour resistance at organ-scale impacted by the importance of the organ for fitness?
Source: BMC Evol Biol. 2018 Dec 6;18:185. doi: 10.1186/s12862-018-1298-7 (PMC6282255; doi:10.1186/s12862-018-1298-7)
Supplement: Supplementary file 2 [file 12862_2018_1298_MOESM2_ESM.odt]

# Supplementary results

## Variability of tumour emergence ()

In our model, we consider two groups of healthy individuals, those who will never develop a tumour () and those who are developing a tumour (). The relative proportion of these two groups is managed by the parameter  which represents the rate of tumour emergence in our population. We assume that formula catches both the genetic susceptibility of the population to cancer and its environmental exposure to carcinogens. 

Here, we vary the value of formula and we propose to compare three scenario:  (i.e., half of the individuals will never develop a tumour),  (i.e., a quarter of the individuals will never develop a tumour),  (i.e., all individuals develop a tumour). 

At the beginning of the simulations, the individuals belonging to the  group are composed by an equal proportion of individuals with the different strategies of tumour resistance. But contrary to the individuals belonging to the group , the variability of fecundity rates of individuals from the group  is not correlated with the variability of tumour initiation rate (which is constant and equal to 0) and is not balanced by the variability of mortality rate (which is constant and equal to ). In other words, decreasing the tumour emergence rate () in our population amounts to decrease the role of cancer on the individual’s mortality. Moreover, we assume that individuals who develop a tumour may have offsprings that never develop a tumour. That way, the evolution of tumour resistance mechanisms between the two groups of individuals (with and without tumour development) is not disconnected.

Figure S1 : Selected resistance strategies and their sensitivity to initial parameters for a high level () an intermediate level () and a low level () of tumour emergence and for a low cost of resistance (). A. Proportion of the different selected resistance strategies for each scenario. B. The Partial Rank Correlation Coefficient (PRCC) between the seven input parameters and the selected tumour resistance strategies in the key organ () and the auxiliary organ (). The significant correlations are in bold(t-test, p>0,01).  The main selective forcesare in red. 

The results show that decreasing the tumour emergence rate () in the population lead to the selection of individuals with a high fecundity rate and the decrease of the selective advantage of high tumour resistance mechanisms (Figure S1).  Interestingly, we observe that the decrease of tumour emergence (from  to ) has mainly the same effect on the evolution of cancer resistance mechanisms than an increase of resistance cost (from  to ). Then, the variability of tumor emergence has no influence on the main selective pressures governing the establishment of cancer resistance mechanisms. Indeed, the cancer-related mortality rate () is the main selective force influencing the evolution of resistance mechanisms in the key organ studied in interaction with the auxiliary organ or studied in isolation, and in the auxiliary organ studied in interaction with the key organ (Figure S1). Moreover, the lethality of tumour stages () is the main selective force influencing the evolution of cancer resistance mechanisms in the auxiliary organ studied in isolation (Figure S1). 

## Relaxing the shape of the resistance-fertility trade-off 

In the main text, we use a Gompertz function to model the trade-off between the tumour initiation rates ( and ) and the fecundity rate of individuals (). The consequence of the sigmoid shape of Gompertz function is that a decrease or an increase of resistance for an individual with an extreme resistance strategy leads to a weaker increase or decrease of fecundity rate than for an individual with an intermediate resistance strategy. 

To test the influence of Gompertz function on our results and relax the assumptions that ensuing its use, we employ a convex function () for which a decrease or an increase of resistance for an individual with a low resistance strategy leads to a stronger increase or decrease of fecundity rate than for an individual with an intermediate resistance strategy or a high resistance strategy (Figure S2). 

This convex function depends on  and  which replace  and  respectively and correspond to the fecundity rate of individuals with tumour initiation rates  and  in the key and the auxiliary organ respectively:

Where  and  are the minimal and maximal fecundity rates experiencing by individuals when the fecundity-resistance trade-off is modelled using a Gompertz function. 

We obtain that  and  are approximately equal to  and  respectively for the extreme values of tumour initiation rates. 

As previously, we assume that the fecundity rate of an individual () depends equally on the resistance strategies of both organs when they are in interaction and exclusively on the resistance strategy of the studied organ when it is in isolation (Table S2).

Using a sigmoid function, we observe five selected cancer resistance mechanisms in the key organ (formula is equal to 0.01, 0.07, 0.08, 0.09 or 0.10) and four in the auxiliary organ (formula is equal to 0.01, 0.08, 0.09 or 0.10). Using the convex function, only the extreme resistance strategies are selected in the key and the auxiliary organs (formulaand formulaare equal to 0.01 or 0.10, Figure S3 A). In the convex version of the resistance-fecundity trade-off, the fecundity rates associated to all the intermediate values of cancer resistance (formulaand formulaare not equal to 0.01 or 0.10) are lower than in the sigmoid version of the trade-off. This can explain that we observe a lower diversity of selected resistance mechanisms in the convex version compared to the sigmoid version. Moreover, the absence of intermediate values leads to a lower power of interpretation. Indeed, we cannot determine the selective pressure influencing the resistance mechanisms selected in the auxiliary organ studied in isolation since mainly the weakest resistance is selected and the lack of variability prevents us from calculating correlations (Figure S3 B). However, the shape of the trade-off doesn’t influence the main selective pressures governing the establishment of cancer resistance mechanisms: the strength of cancer impact on host’s mortality (formula) is the main selective force influencing the resistance mechanisms in the key organ studied in interaction with the auxiliary organ or studied in isolation, and in the auxiliary organ studied in interaction with the key organ (Figure S3 B). 

Figure S2: The convex function formule representing the influence of tumour initiation rates on the fertility rate of individuals for an intermediate cost of resistance (formule). formule represents indifferently the tumour initiation rate in the key organ (formule) or in the auxiliary organ (formule). The dashed line represents the fertility rate of individuals with similar tumour initiation rates in both organs when they are studied in interaction or in only one organ when it is studied in isolation. The grey areas represent the variability of fertility rates when both organs are studied in interaction and have different tumour initiation rates. 

Table S2 : The necessary changes in the fecundity function, the tumour initiation rates and the probability for an organ to transmit cancer to another organ allowing us to compare the resistance strategies selected in organs studied in isolation and in interaction. 

| Scenario | Fecundity function | Tumour initiation rates | Probability to transmit cancer |
| --- | --- | --- | --- |
| Key organ in isolation |  | varies from 0.01 to 0.1  is fixed equal to 0 |  |
| Auxiliary organ in isolation |  | is fixed equal to 0  varies from 0.01 to 0.1 |  |
| Both organs in interaction |  | varies from 0.01 to 0.1  varies from 0.01 to 0.1 | varies from 0.1 to 0.9 |

Figure S3 : A) The selected resistance strategies in the key and the auxiliary organs and B) the sensitivity of strategies to the initial parameters for a convex relationship between the cancer initiation rates and the fecundity rate and with an intermediate cost of resistance (). A. Proportion of the different selected resistance strategies for each scenario. B. The Partial Rank Correlation Coefficient (PRCC) between the seven input parameters and the selected cancer resistance strategies in the key organ () and the auxiliary organ (). The significant correlations are in bold (t-test, p>0,01).  The main selective forces are in red.
